# Comparative Evaluation of Plaque Inhibitory and Antimicrobial Efficacy of Probiotic and Chlorhexidine Oral Rinses in Orthodontic Patients: A Randomized Clinical Trial

**DOI:** 10.1155/2019/1964158

**Published:** 2019-02-20

**Authors:** Shreya Shruti Shah, Supriya Nambiar, Deepa Kamath, Ethel Suman, Bhaskaran Unnikrishnan, Asavari Desai, Sanchit Mahajan, Kushan Kishore Dhawan

**Affiliations:** ^1^Resident, Manipal College of Dental Sciences, Mangalore, Manipal Academy of Higher Education, Manipal 576104, Karnataka, India; ^2^Associate Professor, Department of Orthodontics & Dentofacial Orthopaedics, Manipal College of Dental Sciences, Mangalore, Manipal Academy of Higher Education, Manipal 576104, Karnataka, India; ^3^Professor & Head, Department of Periodontics, Manipal College of Dental Sciences, Mangalore, Manipal Academy of Higher Education, Manipal 576104, Karnataka, India; ^4^Associate Professor, Department of Microbiology, Kasturba Medical College, Mangalore, Manipal Academy of Higher Education, Manipal 576104, Karnataka, India; ^5^Associate Dean & Professor, Department of Community Medicine, Kasturba Medical College, Mangalore, Manipal Academy of Higher Education, Manipal 576104, Karnataka, India; ^6^Reader, Department of Orthodontics & Dentofacial Orthopaedics, Manipal College of Dental Sciences, Mangalore, Manipal Academy of Higher Education, Manipal 576104, Karnataka, India

## Abstract

**Background:**

Antimicrobial agents serve as an effective adjunct in plaque control, and chlorhexidine has been the gold standard. However, the philosophy that natural agents are better for children's oral health is on the rise. Probiotic technology represents a breakthrough approach to maintain oral health by utilizing natural beneficial bacteria commonly found in healthy mouths.

**Aim and Objective:**

To compare efficacy of probiotic and chlorhexidine oral rinses in orthodontic patients.

**Materials and Methods:**

30 healthy patients undergoing fixed orthodontic treatment were randomly selected for the study by block randomization and allocation concealment and were divided into three groups: group a, 0.2% chlorhexidine mouthwash; group b, probiotic mouthwash; and group c, a control group.

**Results:**

Probiotic and chlorhexidine groups had significantly decreased plaque indices as compared to the control group. However, greater improvement was seen in the gingival indices than plaque indices with better results in the probiotic group than the chlorhexidine group. No statistical significance was observed in the streptococcus count of probiotic and chlorhexidine groups at the end of the intervention period.

**Conclusion:**

The comparison of probiotics to chlorhexidine has proven that probiotics are as effective as chlorhexidine as an adjunctive chemical plaque control agent.

## 1. Introduction

Orthodontic tooth movement differs from the physiological tooth movement in that it is a biochemical adaptive response to the application of the orthodontic force with the reorganization of the intracellular and the extracellular matrix, in addition to a change of the local vascularization which in turn leads to the synthesis and the release of arachidonic acid, growth factors, metabolites, cytokines, and various enzymes [1]. Fixed orthodontic appliances are considered to jeopardize dental health due to accumulation of microorganisms that may cause enamel demineralization which manifest as white spot lesions. The complex design of orthodontic bands and brackets may create an ecological environment that facilitates the establishment and growth of cariogenic *streptococcus mutans* strains. Design and surface characteristics of both orthodontic attachment and roughness of the composite influence plaque retention leading to formation of caries [2–5].

*Streptococcus mutans* is most prevalent in pits and fissures of teeth. They colonize abundantly in biofilms of dental plaque in the oral cavity. One of the virulence factors of *S. mutans* in cariogenicity is its ability to attach to the tooth surface and form a biofilm [6]. *S. mutans* grows and synthesizes a dextran capsule which binds it to the enamel and forms a biofilm. From the matrix of the dental plaque, the dextran slime can be depolymerized to glucose for use as a carbon source, resulting in the production of lactic acid within the biofilm (plaque) that decalcifies the enamel and leads to dental caries or bacterial infection of the tooth.

Probiotic is the term currently used to name ingested microorganisms associated with beneficial effects to humans and other animals [7]. They are “live microorganisms which, when administered in adequate amounts, confer a health benefit on the host [8, 9].” Probiotic microorganisms may shape the immune system both at the local and systemic level and have emerged as an alternative way to combat infections. The key event is that, a space in a biofilm that would otherwise be colonized by a pathogen is occupied by harmless microorganisms such as strains of *Lactobacilli* or *Bifidobacteria*. *Bifidobacteria* are the predominant anaerobic bacteria within the intestinal lumen and play a critical role for maintaining equilibrium of the normal gut flora. Many benefits of probiotics on general health have been proposed, among which are decreased susceptibility to infections, reduced allergies and lactose intolerance, lowered blood pressure and lowered serum cholesterol values to name a few [4]. They are capable of influencing many components of epithelial barrier function either by decreasing apoptosis of epithelial cells or increasing mucin production. They act either by inducing host cells to produce peptides or by directly releasing peptides, thus interfering with pathogens and preventing epithelial invasion. Probiotic bacteria compete with invading pathogens for binding sites on epithelial cells, thus boosting the body's defense mechanism [10–13].

There are several different kinds of probiotic organisms. Some of them are enumerated as follows:

- *Lactobacilli*: there are more than 50 species of *Lactobacillus.* They produce natural antibiotics such as lactocidin and acidophilin which enhance immunity. Lactobacilli inhibit the growth of periodontopathogens. Daily consumption reduces the probing depths, resulting in decreased loss of clinical attachment of gingiva to support bone compared to individuals who consume fewer dairy drinks [11].
- *Bifidobacteria:* there are approximately 30 species of *Bifidobacterium.* They produce favourable changes in pH by producing lactic and acetic acid. They also help in increased absorption of minerals.
- *Streptococcus thermophilus*: they help in reduction of lactose intolerance.
- *Enterococcus faecium*: they are very resistant to antibiotics and hence help persistently in the body's defense mechanism.
- *Saccharomyces boulardii*: they are the only probiotic species of yeast.

The main aim of this study was to compare antiplaque, antigingivitis, and anti-*Streptococcus mutans* effectiveness of probiotic and chlorhexidine mouth rinses. This investigation was undertaken with the objectives as to compare the effect of probiotics on the oral health status and gingival status, to evaluate the effect of probiotics on the salivary *S. mutans* colony count and also to compare the effects of probiotic and chlorhexidine mouth rinses on patients undergoing orthodontic treatment.

## 2. Materials and Methodologies

This research (protocol ref no: 14161) was approved by the Institutional Ethics Committee (IEC). The trial was also registered with the Clinical Trials Registry, India, under reference number “201503008658.” The study was performed on orthodontic patients after a written informed consent was taken, as a randomized, controlled trial for a period of 28 days. The sample comprised 30 healthy dentate subjects with power of the sample at 90%. All individuals with full dentition (except third molars) and good/fair oral and general health were included, and individuals with noticeable facial deformities or disfigurement, severe malocclusion, periodontal scaling done within the last 2 months, history of periodontal breakdown, history of parafunctional habits, and history of maxillofacial surgery or jaw injuries were excluded. Patients were randomized to the different groups by the block randomization method. The designated mouthwashes were dispensed to the respective groups after being freshly prepared every week as viability of probiotics is seven days [14]. Group A consisted of the patients who were administered interventional 0.2% chlorhexidine mouthwash [14] (10 ml of chlorhexidine in 10 ml distilled water). Group B consisted of patients who were administered interventional probiotic mouthwash containing 2 × 10^8^ colony-forming units/g [4] (sporlac sachets dissolved in distilled water). Group C consisted of the control group for which no intervention was administered. The interventional groups were administered mouthwashes twice daily after brushing except on the day of the evaluation.

**Figure 1:**
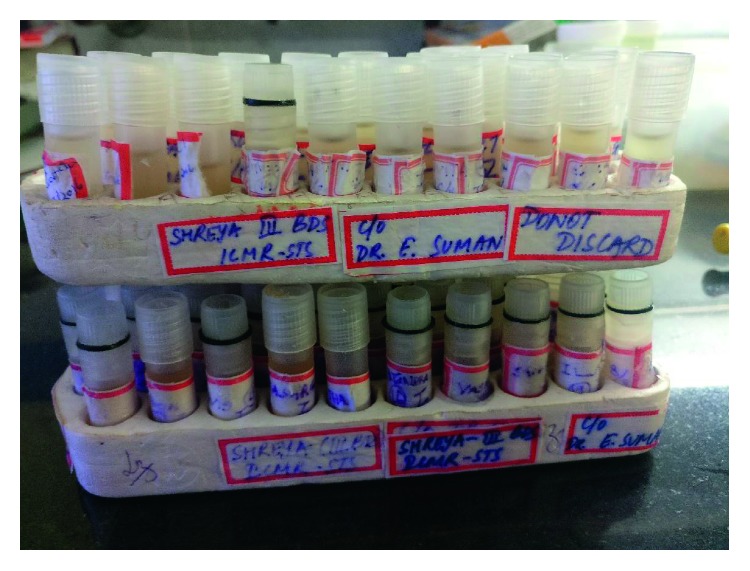
Preserved samples.

**Figure 2:**
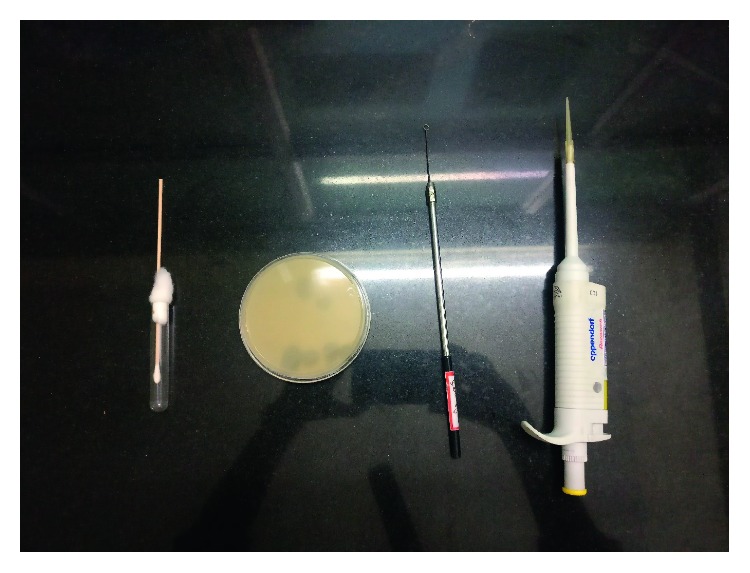
Armamentarium.

**Figure 3:**
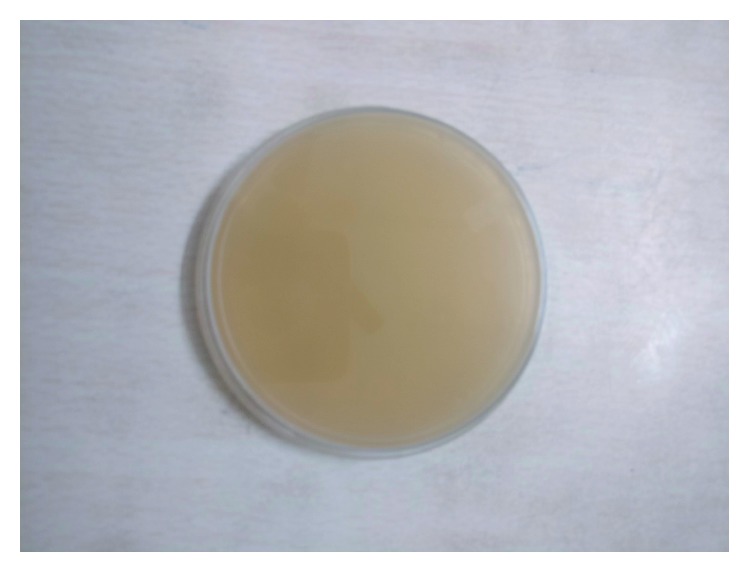
Culture plate.

**Figure 4:**
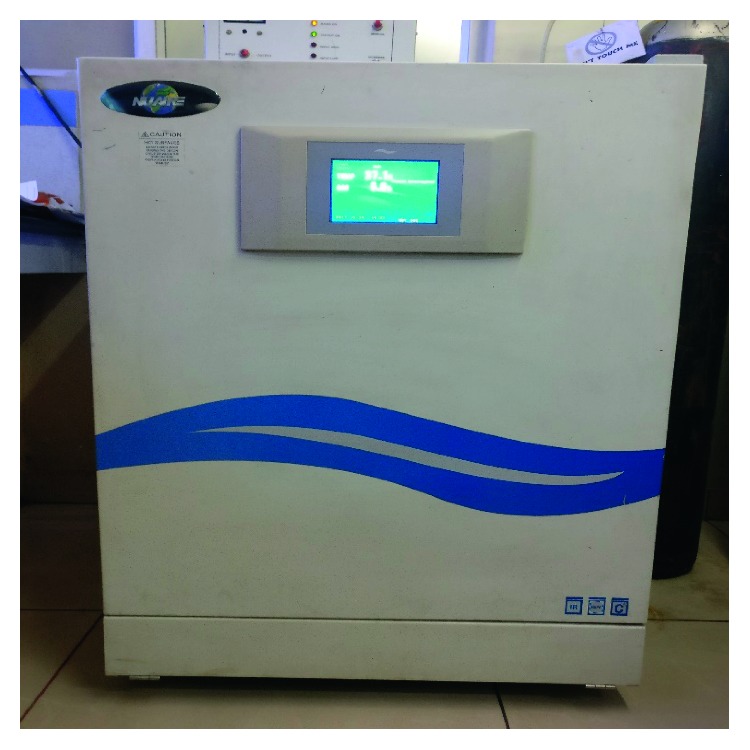
Incubator.

The aforementioned criteria were used:

- For the Plaque Index, Silness and Loe's Plaque Index was assessed for the 4 gingival areas (distofacial, facial, mesiofacial, and lingual surfaces) of only the index teeth (tooth numbers: 16, 12, 24, 36, 32, and 44).
- For the gingival status, Loe and Silness' Gingival Index was assessed for the 4 gingival areas (distofacial, facial, mesiofacial, and lingual surfaces) of only the index teeth (tooth numbers: 16, 12, 24, 36, 32, and 44).
- For streptococcal colony count, the procedure carried out manually by quantitative differential culture at baseline and once every week for four weeks (Figure 1). Saliva was collected in sterile Uricol containers every week. The saliva samples were spread over mitis-salivarius-bacitracin (MSB) culture media (Figure 2), and the colony-forming units per ml (CFU) were measured. The media plates (Figure 3) were then incubated at 37°C in the 5% carbon dioxide incubator (Figure 4) for 48 hours. The *Streptococcus mutans* colonies were identified by morphology under the microscope with ×10 magnification. The colonies were then manually calculated under the naked eye in natural light. The procedure followed was in accordance with the study conducted by Sanchit et al. [15].

Comparison of the gingival and plaque statuses among the three groups was analysed by paired Student's *t*-test using SPSS version 11.0. Comparison of salivary *S. mutans* count among the three groups was done by the Wilcoxon signed-rank test, and *P* value less than 0.05 was considered as statistically significant.

## 3. Observations and Results

For the Plaque Index, the values of both probiotic and chlorhexidine groups showed significant decrease (*P* < 0.05) as compared to the control group The mean Plaque Index value for the chlorhexidine group was 0.88 at baseline and 0.34 at the end of three weeks of intervention. The mean Plaque Index value for the probiotic group was 0.78 at baseline and 0.18 at the end of three weeks of intervention. The mean Plaque Index value for the control group was 1.07 at the baseline and 1.10 at the end of three weeks of intervention (Table 1).

For the Gingival Index, the values of both probiotic and chlorhexidine groups showed significant decrease as compared to the control group. The mean Gingival Index value for the chlorhexidine group was 1.09 at baseline and 0.55 at the end of three weeks of intervention. The mean Gingival Index value for the probiotic group was 1.03 at baseline and 0.16 at the end of three weeks of intervention. The mean Gingival Index value for the control group was 1.24 at the baseline and 1.23 at the end of three weeks of intervention (Table 2).

For streptococcal count, the values of both probiotic and chlorhexidine groups showed significant decrease as compared to the control group. The *S. mutans* colony-forming units per ml (cfu/ml) for the chlorhexidine group was 7.8 × 10^4^ at baseline and 2.7 × 10^3^ at the end of three weeks of intervention. For the probiotic group, it was 7.1 × 10^4^ at baseline and 1.1 × 10^3^ at the end of three weeks of intervention. And for the control group, it was 9.7 × 10^4^ at the baseline and 8.9 × 10^4^ at the end of three weeks of intervention (Table 3).

## 4. Discussion

Chemical plaque control agents are increasingly being used to enhance the effect of mechanical plaque control, though they are not meant as replacement for the latter. Chlorhexidine mouth rinse has been extensively studied and proved to be the most effective antiplaque and antigingivitis agent at present. In spite of this, it has a number of detrimental local side effects like brownish discoloration of teeth and oral mucosa, disturbance of taste, and in severe cases, hypersensitivity and stenosis of the parotid duct. These side effects have created the need for development of alternative antiplaque agents.

Probiotics are currently used widely as a health care adjunct [16]. The antiplaque activity of probiotic mouth rinse may be achieved in several ways, such as reducing bacterial adhesion to the tooth surface, inhibiting growth and proliferation of microorganisms on the tooth surface, inhibiting formation of intercellular plaque matrix, modifying plaque biochemistry to reduce the formation of cytotoxic products, and modifying plaque ecology to a less pathogenic flora [17]. Probiotic mouth rinses utilize the natural commensal bacteria to provide a natural defense system against harmful bacteria. This means that there is no issue of antibiotic resistance and there are no known side effects of probiotics [17]. This 28-day randomized, controlled trial was done to compare the antiplaque, antigingivitis, and anti-*Streptococcus mutans* effectiveness of probiotic and chlorhexidine mouth rinses in a sample population of 30 young adults undergoing orthodontic treatment. This study was designed as a randomized, controlled trial in which 3 different groups of subjects were tested. Groups A and B received probiotic and chlorhexidine intervention, respectively, whereas group C was the control group which received no intervention. The subjects were segregated into these 3 groups in a randomized order. This study demonstrated that the probiotic and chlorhexidine groups had significantly decreased plaque indices as compared with the control group at the end of the intervention period as found by Harini et al. [13]. However, greater improvement was seen in gingival indices compared to plaque indices, and the probiotic group showed better results than the chlorhexidine group.

The study also reinforced that, at the end of the intervention period, there was significant reduction in the *Streptococcus* count in the probiotic and chlorhexidine groups as compared with the control group in agreement with other studies [4, 5, 16]. It was also observed that there was a statistically significant difference in the *Streptococcus* count in the probiotic and chlorhexidine groups at the end of the intervention period as concluded by Jose et al. [5]. From this study, it was observed that probiotic mouth rinse had a significant inhibitory effect on plaque accumulation and gingival inflammation. Thus, it can be proposed that probiotic mouth rinse had a potential therapeutic value in reducing plaque accumulation and gingivitis. The findings of this clinical study are in agreement with a previous study conducted by Harini et al. [13]. However, the results of the two studies cannot truly be compared as the other study was conducted among children and not in adults. Vitality of *Bifidobacteria* at room temperature is seen to be seven days as found by Sarvari et al. [17]. Viability of probiotic bacteria in the mouth rinse in this study is questionable as greater improvement of the Plaque Index for chlorhexidine as compared to probiotics is seen at the end of second week of intervention (Table 1). This could be due to decrease in the viability of probiotic bacteria as the week progresses. Also, the optimal daily dose is not yet established as probiotics do not colonize the oral cavity permanently. The dilution of probiotics used in this study was the same as that used in the study conducted by Cildir et al. [4].

However, it is possible that a higher concentration of probiotics may be more effective. It is also possible that a combination of multiple probiotic strains could be even more effective. Further studies on adolescent patients with fixed orthodontic appliances constitute a very suitable group for risk patients concerning enamel remineralization with probiotic supplements. There are no long-term studies available on the effect of probiotic bacteria on the oral microflora as yet. If successful, the probiotic home-care intervention may be a cost-effective alternative to a professional topical programme for oral health care during orthodontic treatment.

## 5. Conclusion and Summary

This clinical trial has helped us to study the effect of probiotics on the oral health status and gingival status and also to evaluate the effect on salivary *S. mutans* colony count of patients undergoing orthodontic treatment. The comparison of probiotics to chlorhexidine has proven that probiotics are as effective as chlorhexidine as an adjunctive chemical plaque control agent. The probiotic and chlorhexidine groups had significantly decreased plaque indices as compared with the control group at the end of the intervention period. However, greater improvement was seen in the gingival indices compared to plaque indices with the probiotic group showing better results than the chlorhexidine group. The study also reinforced that, at the end of intervention period, there was significant reduction in the *Streptococcus* count in the probiotic and chlorhexidine groups. The findings suggest that the probiotic mouth rinse is effective in reducing plaque accumulation and gingival inflammation. Daily use of probiotic mouth rinse could reduce the levels of *Streptococcus mutans* in the saliva in orthodontic patients with fixed appliance. Therefore, the probiotic mouth rinse has a potential therapeutic value, and use of powdered probiotic sachets mixed in water can be used as a safer and cheaper alternative oral health care regimen which will benefit public health policies for larger interest. Further long-term studies are recommended to determine its efficacy for prevention of demineralization and development of caries during orthodontic treatment.

## Figures and Tables

**Table 1:** Comparison of the mean plaque value scores between the groups.

Plaque Index (PI)	Chlorhexidine	Probiotics	Control
	Mean ± SD	Mean ± SD	Mean ± SD
1^st^ week	0.88 ± 0.46	0.78 ± 0.34	1.07 ± 0.21
2^nd^ week	0.70 ± 0.53	0.66 ± 0.31	1.45 ± 0.43
*P* value	0.03	0.05	0.25

2^nd^ week	0.70 ± 0.53	0.66 ± 0.31	1.45 ± 0.43
3^rd^ week	0.52 ± 0.45	0.52 ± 0.32	1.29 ± 0.42
*P* value	0.12	0	0.76

3^rd^ week	0.52 ± 0.45	0.52 ± 0.32	1.29 ± 0.42
4^th^ week	0.34 ± 0.44	0.18 ± 0.22	1.10 ± 0.52
*P* value	0	0.07	0

1^st^ week	0.88 ± 0.46	0.78 ± 0.34	1.07 ± 0.21
4^th^ week	0.34 ± 0.44	0.18 ± 0.22	1.10 ± 0.52
*P* value	0	0.13	0.87

Statistically significant at *P* < 0.05.

**Table 2:** Comparison of the mean gingival value scores between the groups.

Gingival Index (GI)	Chlorhexidine	Probiotics	Control
	Mean ± SD	Mean ± SD	Mean ± SD
1^st^ week	1.09 ± 0.48	1.03 ± 0.58	1.24 ± 0.12
2^nd^ week	0.96 ± 0.43	0.80 ± 0.49	1.35 ± 0.27
*P* value	0.10	0	0.11

2^nd^ week	0.96 ± 0.43	0.80 ± 0.49	1.35 ± 0.27
3^rd^ week	0.84 ± 0.53	0.54 ± 0.37	1.31 ± 0.46
*P* value	0.09	0.05	0.59

3^rd^ week	0.84 ± 0.53	0.54 ± 0.37	1.31 ± 0.46
4^th^ week	0.55 ± 0.52	0.16 ± 0.18	1.23 ± 0.10
*P* value	0.01	0.11	0.66

1^st^ week	1.09 ± 0.48	1.03 ± 0.58	1.24 ± 0.12
4^th^ week	0.55 ± 0.52	0.16 ± 0.18	1.23 ± 0.10
*P* value	0	0.39	0.54

Statistically significant at *P* < 0.05.

**Table 3:** Comparison of the median streptococcal colony count scores between the groups.

Streptococcal count (SC)	Chlorhexidine	Probiotics	Control
	Median (IQR)	Median (IQR)	Median (IQR)
1^st^ week	7.8 × 10^4^ (2 × 10^3^ – 1.1 × 10^5^)	7.1 × 10^4^ (6 × 10^3^ – 1.1 × 10^5^)	9.7 × 10^4^ (3.7 × 10^3^ – 1.6 × 10^5^)
2^nd^ week	4 × 10^4^ (1.6 × 10^3^ – 3.8 × 10^4^)	7.8 × 10^3^ (2.2 × 10^3^ – 6.3 × 10^3^)	8.6 × 10^4^ (2.5 × 10^3^ – 1.5 × 10^5^)
*P* value	0.28	0.72	0

2^nd^ week	4 × 10^4^ (1.6 × 10^3^ – 3.8 × 10^4^)	7.8 × 10^3^ (2.2 × 10^3^ – 6.3 × 10^3^)	8.6 × 10^4^ (2.5 × 10^3^ – 1.5 × 10^5^)
3^rd^ week	1.5 × 10^4^ (5.5 × 10^2^ – 4.1 × 10^3^)	3.1 × 10^3^ (10^3^ – 5.7 × 10^3^)	9.2 × 10^4^ (2.3 × 10^3^ – 1.5 × 10^5^)
*P* value	0.10	0.06	0

3^rd^ week	1.5 × 10^4^ (5.5 × 10^2^ – 4.1 × 10^3^)	3.1 × 10^3^ (10^3^ – 5.7 × 10^3^)	9.2 × 10^4^ (2.3 × 10^3^ – 1.5 × 10^5^)
4^th^ week	2.7 × 10^3^ (5.2 × 10^2^ – 2.9 × 10^3^)	1.1 × 10^3^ (6.4 × 10^2^ – 1.4 × 10^3^)	8.9 × 10^4^ (2.2 × 10^3^ – 1.5 × 10^5^)
*P* value	0.30	0.28	0

1^st^ week	7.8 × 10^4^ (2 × 10^3^ – 1.1 × 10^5^)	7.1 × 10^4^ (6 × 10^3^ – 1.1 × 10^5^)	9.7 × 10^4^ (3.7 × 10^3^ – 1.6 × 10^5^)
4^th^ week	2.7 × 10^3^ (5.2 × 10^2^ – 2.9 × 10^3^)	1.1 × 10^3^ (6.4 × 10^2^ – 1.4 × 10^3^)	8.9 × 10^4^ (2.2 × 10^3^ – 1.5 × 10^5^)
*P* value	0.10	0.96	0

Statistically significant at *p* < 0.05.

## Data Availability

The data used to support the findings of this study are included within the article.

## Additional Points

*Relevance/Importance of the Study*. This investigation was undertaken to compare the effect of probiotics on the oral health status and gingival status of patients undergoing orthodontic treatment. It was also done to evaluate the effect of probiotics on the salivary *S. mutans* colony count of patients undergoing orthodontic treatment and to compare and evaluate effects of probiotic and chlorhexidine mouth rinses. It was observed that daily use of probiotic mouth rinse could reduce the levels of *Streptococcus mutans* in the saliva in orthodontic patients with fixed appliance. Therefore, the probiotic mouth rinse has a potential therapeutic value, and use of powdered probiotic sachets mixed in water can be used as a safer and cheaper alternative oral health care regimen which will benefit public health policies for larger interest.
